# Postoperative thyroid crisis in an 11-year old male with McCune-Albright syndrome and atypical triiodothyronine hyperthyroidism: A case report

**DOI:** 10.1097/MD.0000000000028928

**Published:** 2022-03-04

**Authors:** Jingen Hu, Caibao Hu

**Affiliations:** a Department of Orthopedics, the First Affiliated Hospital, School of Medicine, Zhejiang University, No. 79 Qingchun Road, Hangzhou 310003, Zhejiang, China; b Intensive Care Unit, Zhejiang Hospital, 12 Lingyin Road, Hangzhou 310013, Zhejiang, China.

**Keywords:** Café-au-lait macules, hyperthyroid, McCune-Albright syndrome, polyostotic fibrous dysplasia

## Abstract

**Rationale:**

McCune-Albright syndrome (MAS) is a rare heterogeneous clinical disease caused by sporadic, somatic, and postzygotic mutations. Thyroid crisis is even rare in patients with MAS, and we report the clinical outcomes of the first case of a MAS patient with atypical triiodothyronine (T3) hyperthyroidism who developed thyroid crisis after orthopedic surgery.

**Patient concerns:**

The patient with MAS and atypical T3 hyperthyroidism was an 11-year-old man who had undergone surgery for a right femur fracture and shepherd bending deformity. His main symptoms were dizziness, nausea, and vomiting with elevated body temperature because of developed thyroid crisis. Thyroid function tests showed high T3 and remarkably high free T3 levels, and remarkably increased thyrotropin level, but unchanged thyroxine and free thyroxine levels.

**Diagnosis:**

The patient was diagnosed with postoperative thyroid crisis following surgery for a right femur fracture, shepherd bending deformity, and MAS with atypical T3 hyperthyroidism.

**Interventions:**

Propranolol was intravenously administered. The therapy included intravenous hydrocortisone, a saturated solution of potassium iodine and propylthiouracil, and continuous physical cooling.

**Outcomes:**

The patient was discharged after achieving a stable condition with normal thyroid and liver function after surgery because of active anti-thyroid crisis treatment.

**Lessons:**

The operation of such patients should focus on the pre-operative heart rate, platelet level, and thyroid hormone levels. Abnormal values should be adjusted to the normal range, and such patients should achieve complete hemostasis and transfuse with blood following surgery anemia.

## 1 Introduction

McCune-Albright syndrome (MAS), also known as Albright syndrome, is characterized by Café-au-lait macules (CALMs), multiple ossifying fibrous dysplasia, and autonomic and hyperfunctional endocrine diseases including acromegaly, Cushing syndrome, and hyperthyroidism. It is associated with somatic activating mutations in the G-protein alpha stimulatory subunit (Gsa subunit) gene (GNAS).^[1,2]^ Previous studies^[3,4]^ have described 2 patients with MAS who developed thyroid toxic crisis and was associated with surgery, including in conjunction with non-autoimmune hyperthyroidism. To the best of our knowledge, thyroid crisis has not been reported in MAS patients with atypical triiodothyronine (T3) hyperthyroidism. Here, we report the treatment experience of a patient with MAS who developed a thyroid crisis after surgery with atypical T3 hyperthyroidism.

## 2 Case report

An 11-year old male patient with no history of consanguinity in the family presented with large patches of cutaneous pigmentation since birth and multiple bony deformities developing over the last 4 years.

**Figure 1:**
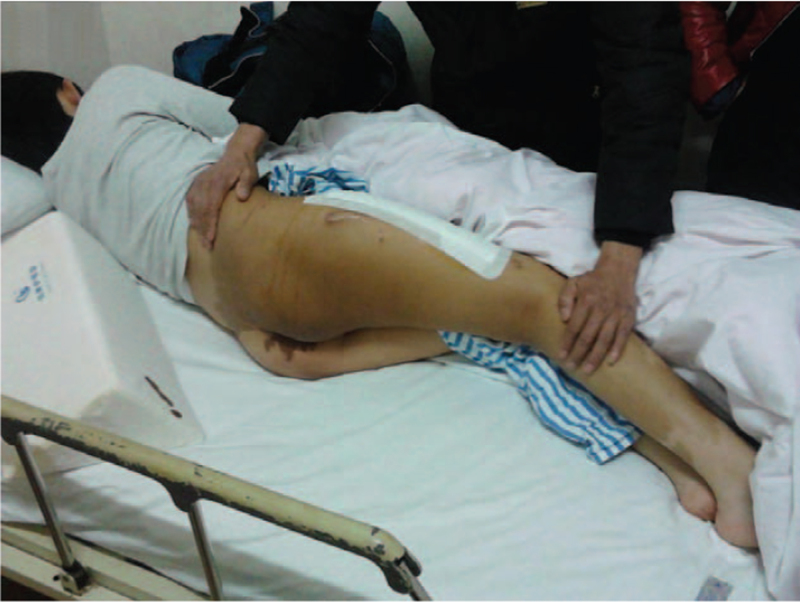
Giant CALMs on the right lower back, buttock and the right leg were observed. CALMs = Café-au-lait macules.

On examination, giant CALMs were observed on the right lower back, buttock, and right leg (Fig. 1). CALMs were observed respecting the midline and had serrated margins (coast of Maine) in comparison with smooth margins (coast of California) in neurofibromatosis. CALMs were observed along the midline on the right side of the abdomen, with serrated margins (coast of Maine), unlike the smooth margins (coast of California) of neurofibromatosis.

**Figure 2:**
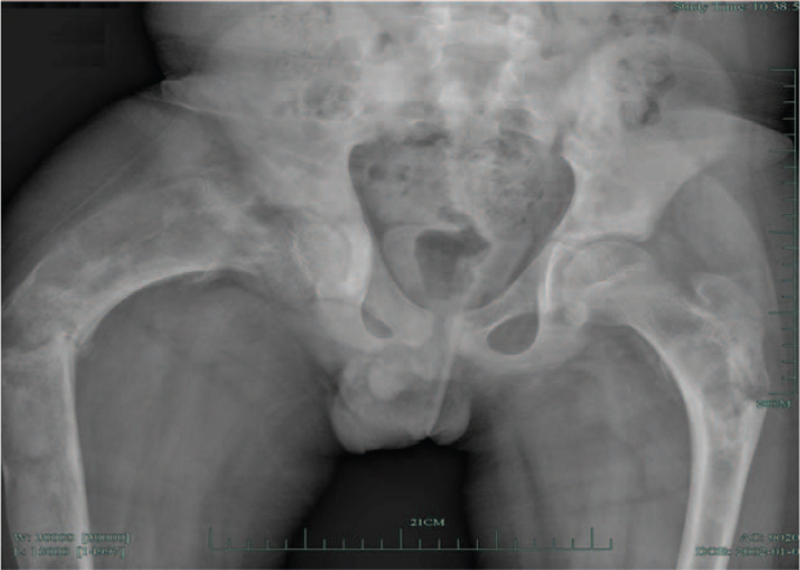
X-rays of both the femor showed extensive bowing deformities and right femur fracture.

The patient gradually developed deformities in both lower limbs and was unable to walk without assistance. The patient often had bony pain and multiple pathological malhealed fractures that healed at a normal rate. The patient had extensive bow deformities in both lower limbs (Fig. 2). The patient underwent several internal fixation operations due to repeated fractures of the long bones. In addition, the intelligence of patients is the same as that of healthy people.

Radiolucent and radiodense areas existed in all affected long bones, accompanied by bone marrow replacement and loss of cortical-medullary differentiation. All long bones showed signs that the old fractures had healed. Bilateral femurs were involved in progressive varus changes, resulting in a characteristic “shepherd bend” deformity. Computed tomography revealed fibrous dysplasia of the sacrum, bilateral femora, iliac, and right tibia. The skull X-ray showed hardening of the skull base and the diploae widening of the occipital region. Sclerosis of the occipital region were evident.

Bone biopsy showed narrow, curved, “fishhook” bone trabecular tissue dotted with fibrous tissue. Biochemical examination showed that the serum calcium level was within the reference range, but the level of serum alkaline phosphatase was significantly increased: 566 mg/dL (reference range: 40–150 mg/dL) and human growth hormone: 10.60 ng/mL (reference range: 0–0.8 mg/dL). Of note, pre-operative thyroid function tests showed serum concentrations of total thyroxine of 162.5 nmol/L (normal 55.47–161.25 nmol/L), T3 of 3.68 nmol/L (normal 1.02–2.96 nmol/L), thyrotropin of 0.020 mlU/L (normal 0.380–4.340 mlU/L), free thyroxine of 21.09 pmol/L (normal 10.45–24.38 pmol/L), free T3 of 9.18 pmol/L (normal 2.77–6.31pmol/L). Tests for anti-thyroglobulin antibody and anti-thyroid peroxidase autoantibody were negative, and no other endocrine abnormalities (parathyroid hormone, cortisol, corticotropin, follicle-forming hormone, luteinizing hormone, estradiol, progesterone, testosterone, prolactin) were found in the patient. Visual examination and palpation of the thyroid were normal, and no abnormalities were found by ultrasound, including nodules and cysts.

He had no symptoms of sweating, tachycardia, or irritability and no history of diarrhea. The patient showed atypical T3 hyperthyroidism, had no obvious symptoms of hypermetabolism, and had no manifestations of hyperthyroidism. The patient had no signs of exophthalmos. Routine blood tests and coagulation function tests were normal before the operation. The diagnosis of MAS and atypical T3 hyperthyroidism was confirmed based on clinical, radiological, biochemical, and biopsy results.

**Figure 3:**
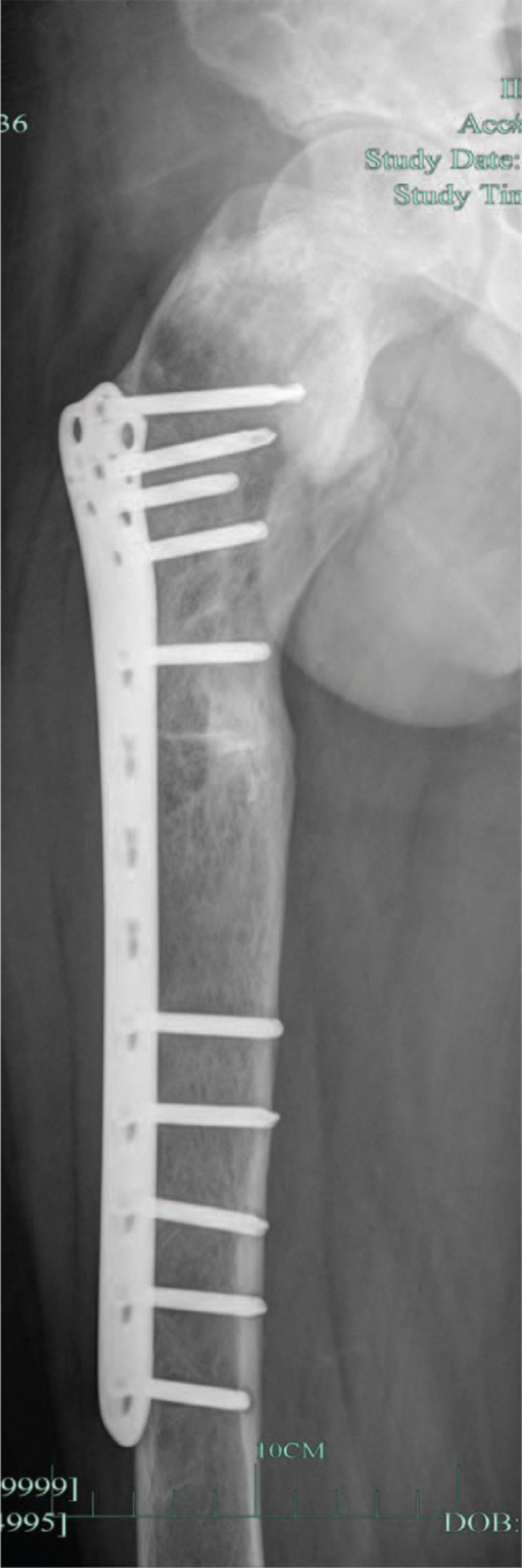
After right femoral curettage + grafting + osteotomy fixation.

The patients were treated with curettage of right femur, bone grafting and osteotomy and fixation (Fig. 3) under general anesthesia. First day after surgery, the patient complained of dizziness, nausea, and vomiting of gastric contents once with his body temperature recorded at 38.4°C. On the second postoperative day, the patient's temperature reached as high as 39.0°C, pulse increased to 150/min, and blood pressure was maintained at 100 to 120/50 to 60 mm Hg. Our endocrinologist concluded that a thyroid crisis could not be excluded. The hemoglobin level of the patients decreased directly from pre-operative 145 g/L to postoperative 92 g/L. The patient did not take any antiplatelet drugs. Significant perioperative blood loss is more likely to cause thyroid crisis.

The drugs were used with particular caution because of cardiac arrhythmias and angina pectoris. Informed consent for DNA analysis was not obtained from thyroid biopsy and blood samples.

Propranolol was administered intravenously (10 mg every 6 hours). The therapy included intravenous hydrocortisone (100 mg every 6 hours), 5 drops orally every 8 hours of a saturated solution of potassium iodine, 200 mg of propylthiouracil orally every 6 hours, and continuous physical cooling. On the third day after surgery, the body temperature dropped to 36.8°C and the heart rate decreased to 90 to 100/min. The symptoms of nausea and vomiting disappeared. The dose of hydrocortisone was gradually decreased to 0.

Simultaneously, the patient was required to avoid iodine-containing food and to undergo routine blood tests every week; thyroid function was tested every 3 to 4 weeks, and liver function was tested every 2 weeks. Tenth day after surgery, no abnormal findings were observed. The incision healed without hemodialysis. The patient's condition was stable with normal thyroid and liver function. Subsequently, propranolol was changed to 10 mg oral bid, and potassium iodide was stopped. The patient was discharged after the surgery and was in stable condition after operation.

## 3 Discussion

Postoperative thyroid crisis as a complication of thyroidectomy was once quite common, but is now rarely seen, let alone in MAS. No report has focused on postoperative thyroid crisis in patients with MAS and atypical T3 hyperthyroidism.

The factors that precipitate postoperative thyroid crisis are not always identifiable or can only be identified in retrospect. Significant perioperative blood loss may be the most common precipitating event in the patient, and we believe that the occurrence of thyroid crisis in our patient was related to the amount of perioperative blood loss. Perioperative blood loss included intraoperative and postoperative bleeding. The increased perioperative blood loss in MAS patients undergoing orthopedic surgery was previously thought to be due to increased blood vessels in the dysplastic bone.^[5]^ Another study observed^[6]^ increased bleeding tendency and abnormal platelet function in 3 children with MAS. They speculated that platelet dysfunction is also a cause of excessive blood loss. Platelet dysfunction is another event that initiates a postoperative thyroid crisis. We think our patient had both factors, and MAS is a rare multisystem disorder. The disorder is caused by a postzygotic somatic mutation in the GNAS1 gene on the chromosome 20q13-13.29,^[7,8]^ which encodes the alpha subunit of the stimulating G protein (Gsa). This mutation leads to the accumulation of intracellular cAMP and the activation of cAMP-dependent receptors (such as corticotropin, growth hormone, thyrotropin, luteinizing hormone, and follicle-forming hormone receptors), which enhance the function of related hormones in some target organs and target cells, such as increased melanin secretion by melanocytes, resulting in skin pigmentation. It can also promote the accumulation of cAMP in platelet cells and lead to platelet dysfunction, which in turn leads to postoperative thyroid crisis. Because of platelet dysfunction, there was more bleeding during and after the operation, which led to postoperative thyroid crisis in our case.

Since thyroid crisis is a train of physiological derangements from which it is difficult to retrieve the patient, avoidance of the circumstances that lead to it, or at least recognition of the prodromal signs, is the most important. Thus, the emphasis is on prevention. It can be said that the patient with MAS, if they have not reached the stage of storm or impending storm, can be controlled with methods available today. Recognizing the potential and prompt action to thwart it are cardinal objectives.

Adequate pre-operative preparation of patients with MAS is the best preventive measure against thyroid crisis. With regard to platelet dysfunction in patients with MAS, we emphasize the need to evaluate platelet function during surgery, and the disappearance of fine tremor and decline of the resting heart rate to 75 to 85 beats/min are important criteria for adequate pre-operative preparation of patients with MAS. As the patient is a child, with a higher heart rate than the adult, the children's heart rate before operation at 100 beats/min or more may be the normal heart rate. Therefore, a heart rate of 100 beats/min or above is not necessarily one of the causes of thyroid function hyperfunction after surgery in our patient, and thyroid hormone levels during the operation were normal.

The case study presented here suggests that in order to avoid thyroid crisis postoperatively, the patient's heart rate in the low 90 beats/min before operation, except for children or adolescents, is recommended for patients with MAS pre-operatively. In the pediatric population, the development of thyroid storm after surgery in patients with recessive hyperthyroidism has not previously been reported as a postoperative complication. Thyroid function may only reflect the underlying hyperthyroidism state; however, a thyroid storm is a clinical diagnosis. Our case shows that in pediatric patients with a higher risk of thyroid disease, a thyroid storm may occur even after exposure to the same patient's previously well-tolerated pre-disposing factors and hidden hyperthyroidism before surgery. High-risk children with thyroid dysfunction, especially those with an increased incidence of fractures in patients with endocrine diseases of MAS,^[9]^ should be actively screened and monitored for thyroid storm before and after surgery.

In addition, recent genotype-phenotypic correlation studies^[10]^ have shown that the advantage of R201H variation may be of great significance for the development of targeted therapies. Targeted therapy and gene therapy for MAS and its hyperfunctional endocrine diseases are also promising treatment directions in the future.

## Author contributions

**Conceptualization:** Jingen Hu.

**Data curation:** Jingen Hu.

**Formal analysis:** Jingen Hu.

**Funding acquisition:** Jingen Hu.

**Investigation:** Jingen Hu.

**Resources:** Jingen Hu.

**Supervision:** Caibao Hu.

**Writing – original draft:** Jingen Hu.

**Writing – review & editing:** Caibao Hu.
